# Increased prevalence of obstructive sleep apnea in women diagnosed with endometrial or breast cancer

**DOI:** 10.1371/journal.pone.0249099

**Published:** 2021-04-07

**Authors:** Ayey Madut, Veronika Fuchsova, Hong Man, Shabeel Askar, Ritu Trivedi, Elisabeth Elder, Christine L. Clarke, Gerard Wain, Alison Brand, Anna DeFazio, Terence Amis, Kristina Kairaitis

**Affiliations:** 1 Ludwig Engel Centre for Respiratory Research, The Westmead Institute for Medical Research, Sydney, Australia; 2 Westmead Clinical School, Faculty of Medicine and Health, The University of Sydney, Sydney, Australia; 3 Breast Cancer Institute, Westmead Hospital, Sydney, Australia; 4 Centre for Cancer Research, The Westmead Institute for Medical Research, Sydney, Australia; 5 Department of Gynecological Oncology, Westmead Hospital, Sydney, Australia; 6 Sydney West Translational Cancer Research Centre, Sydney, Australia; 7 Department of Respiratory and Sleep Medicine, Westmead Hospital, Sydney, Australia; University of Rome Tor Vergata, ITALY

## Abstract

**Background:**

Epidemiological studies demonstrate associations between obstructive sleep apnea (OSA) and cancer incidence and mortality. The aim of this study was to measure OSA in women with breast (BC) or endometrial cancer (EC) and associations with clinico-pathological tumor variables.

**Methods and findings:**

In a cross sectional study, women with BC (12 months) or EC (3 months) post-diagnosis were recruited from cancer clinics. We collected demographic, anthropometric data, cancer stage, grade, histopathology and history of cancer treatment and all subjects had in-laboratory polysomnography. Sleepiness was assessed with the Epworth Sleepiness Scale (ESS). We compared anthropometric and polysomnographic data between cancer groups (unpaired t-tests), and assessed relationships between cancer characteristics and OSA variables (Fishers exact test). There were no significant differences between average age (BC:59.6±8.7 years(n = 50); EC:60.3±7.7 years(n = 37)), or ESS score (BC:6.4±4.4; EC 6.8±4.7; mean±SD; all p>0.2), however, BMI was higher in EC (BC: 29.7±7.9kgm^-^2; EC: 34.2±8.0 kgm^-^2; p<0.05). BC had longer sleep latency (BC:31.8±32minutes; EC:19.3±17.9 minutes), less Stage 3 sleep (BC:20.0±5.2%; EC:23.6±8.2%) and more REM sleep (BC:21.1±6.9%; EC: 16.6±5.7%), all p<0.05. EC had lower average awake and asleep oxygen saturation levels (BC: 95.6±1.3%; EC: 94.6±1.9% [awake]: BC: 94.8±2.1%; EC: 93.3±2.4% [asleep]; both p<0.05). Apnea-Hypopnea Index (AHI) (BC: 21.2(7.3–36.9) events/hr; EC: 15.7 (10–33.5) events/hour (median (interquartile range)) was not different p = 0.7), however, 58% and 57% of women with BC and EC respectively, had an AHI>15 events/hour. In this small sample size group, no significant associations (all p>0.1) were detected between OSA metrics and clinico-pathological tumor variables.

**Conclusion:**

In postmenopausal women with breast or endometrial cancer there is high prevalence of OSA, with no association with specific tumor characteristics detected. Recognition of the high prevalence of OSA in women with cancer is important to recognise as it may impact on surgical risk and quality of life.

## Introduction

A number of epidemiological studies have reported increased mortality rates in cancer patients with co-morbid obstructive sleep apnoea (OSA) [1, 2]. Across a number of cancer types, individuals with severe OSA have an adjusted hazard ratio of 4.8 for dying as a consequence of cancer progession [1]. When intermittent hypoxia, a physiological consequence of recurrent upper airway obstruction during sleep, is considered, this relationship becomes even stronger with an estimated hazard ratio of 8.6 for patients with severe levels of nocturnal oxygen desaturation [1]. In an Australian population study, moderate to severe OSA was associated with an adjusted cancer mortality odds ratio (OR) of 3.4, and a cancer incidence OR of 2.5 [2].

Most reports to date have focused on retrospective data across a variety of cancer types. Prospective studies examining associations between OSA and specific cancers are sparse. However, studies have reported associations between sleep disordered breathing and cutaneous melanoma [3, 4], while another has reported a high prevalence of OSA in lung cancer patients [5].

For women with cancer in particular, little attention has been paid to potential OSA-cancer associations, and even the prevalence of OSA in these cohorts is mostly unknown. For breast cancer patients, one prospective study of 83 breast cancer patients found a prevalence rate for moderate-severe (apnea hypopnea index >15 events/hour) OSA of 13%, although they could find no link between markers of cancer aggressiveness and sleep disordered breathing [6]. This lack of information is particularly problematic given obesity is a known major risk factor for both OSA and a number of cancers in women, in particular, endometrial cancer and post-menopausal breast cancer [7]. In addition, in these female cohorts, there are also potential sleep-disorder-promoting interactive factors at play, some of which are linked to cancer risk factors themselves (eg age, menopause, obesity). For some female cancers, risk factors shared with OSA are particularly strong (eg obesity and endometrial cancer [7–9]).

In the present study, the prevalence of OSA in women with breast and endometrial cancer was measured using prospective, in-laboratory, polysomnography data. A secondary aim was to test for potential linkages between the severity of sleep disordered breathing and tumour histopathology markers, particularly those associated with tumour aggressiveness.

## Materials and methods

All subjects were prospectively recruited from cancer clinics at Westmead Hospital between May 2016-March 2019. Subjects were approached in person by the researcher in the clinic and asked if they would participate in a study regarding sleep and cancer. All subjects gave written informed consent and the protocol was approved by the Western Sydney Local Health District Human Research Ethics Committee.

### Subjects

All women recruited had a previous diagnosis of endometrial or breast cancer. Prior to recruitment, all had completed a course of cancer therapy that included surgery, and/or radiotherapy and/or chemotherapy. Women with endometrial cancer (n = 37) were recruited 8–12 weeks following surgical treatment (hysterectomy), while women diagnosed with breast cancer (n = 51) were recruited 12 months after completion of primary treatment. Breast cancer patients on anti-oestrogen therapy, including tamoxifen or aromatase inhibitors, were included in the study. Data collected included other comorbidities, as well as primary (eg surgery, radiotherapy, chemotherapy) and ongoing treatment regimens. Patients were not included if they had previously been diagnosed with OSA.

### Anthropometric data

Age was recorded and anthropometric data including height, weight and neck circumference were measured at the time of recruitment.

### Cancer histopathology

Information regarding histopathology and cancer treatment regimes was collected from the patient’s medical record. For women with breast cancer, this included: Nottingham tumor grade [10], tumour size and stage, cancer subtype, estrogen or progesterone receptor staining, staining for Ki67 (a marker of cell proliferation), human epidermal growth factor 2 (HER2), and mitotic index. For women with endometrial cancer, data were collected regarding histological subtype (endometrioid, serous, mixed or clear cell), grade, FIGO stage [11], and staining for estrogen receptor, progesterone receptor and the tumor suppressor TP53.

### Assessment of sleepiness and OSA risk

Subjects completed an Epworth Sleepiness Scale (ESS) [12] questionnaire to document subjective sleepiness. A STOP BANG questionnaire [13] was completed to assess risk of OSA. A STOP BANG score greater than 3 was considered to indicate OSA risk [14].

### Polysomnography

All subjects underwent full in-laboratory overnight polysomnography with monitoring of EEG, EOG, chin, diaphragm and pre-tibial EMG, nasal pressure, oro-nasal thermistor signals, sound (snoring), thoracic and abdominal plethysmography and oximetry. Studies were scored according to American Academy of Sleep Medicine criteria [15]. Values for total sleep time, sleep efficiency, sleep onset latency, wake after sleep onset (minutes, WASO), sleep stage (% sleep time), apnea hypopnea index (AHI, events per hour sleep), respiratory disturbance index (RDI, events per hour of sleep), central apnea index (events/hour), average oxygen saturation level (SpO2 awake) obtained in the upright awake seated position for 3 minutes before sleep, average oxygen saturation during sleep (SpO2 sleep), oxygen desaturation index (ODI3%), and total sleep time with an oxygen saturation less than 90% (mins, TST<90%) were obtained for each subject.

### Statistical analysis

Sample size and power calculation: The study sample size was not based on a power calculation and therefore this study should be considered exploratory only.

This study was designed a cross-sectional observational study with the main outcomes based on comparisons between two exclusively female cancer-type patient groups. Group mean and standard deviation, or median and interquartile ranges for non-normally distributed data were calculated as appropriate. Anthropometric and sleep data for each female cancer patient cohort were compared using unpaired two-tailed t-tests. Data that were not normally distributed (AHI, ODI, TST<90%, nadir SpO2(%)) were compared using an independent samples median test with Yates continuity correction. P<0.05 was considered significant.

#### Relationship between indices of sleep disordered breathing and cancer histopathological characteristics

Exploratory analyses were performed to assess for relationships between markers of sleep disordered breathing severity and histopathological markers. Two categorical sleep-disordered-breathing-severity metric based sub-groups for each cancer group were constructed using (separately) values ≥ or < the median values for AHI, RDI, ODI (3%) and TST<90% contained in the data set for that cancer cohort (ie a total of 2x4 sub-groups). For endometrial cancer patients, a median AHI of 15.7 events per hour, ODI of 10 events per hour, RDI of 18.9 events per hour and TST< 90% of 2.4% were used. For breast cancer patients, categories were assigned using a median AHI of 21.5 events per hour, ODI of 7.3 events per hour, RDI of 23.8 events per hour and TST<90% of 0.5%. Relationships between these categorical sleep disordered-breathing severity sub-groups and histopathological markers were compared using Fisher’s exact test. For the women with breast cancer, relationships between the categorical variables of sleep apnea severity and; Ki67 staining (≤15%, >15% staining), mitotic index, estrogen receptor staining, progesterone receptor staining, HER2 receptor staining, tumour grade were examined. For endometrial cancer the relationships between the categorical variables of sleep apnea severity and tumor grade and FIGO stage were examined. There was insufficient data to analyse the relationships with estrogen and progesterone receptors or TP53, as all cancers stained for estrogen receptor, only one endometrial cancer was negative for the progesterone receptor and only 7 stained for TP53. As there were few stage III and IV endometrial cancers, analysis was confined to Stage I, and Stage II-IV tumours.

## Results

A total of 51 women diagnosed with breast cancer and 37 diagnosed with endometrial cancer consented to participate in the study and were included. One breast cancer patient only slept for 90 minutes during the polyomnography study and therefore all their data was excluded from the analysis.

### Anthropometric data

Anthropometric and ESS questionnaire data are shown in Table 1. Both groups of women were predominantly middle aged, and there was no significant difference in mean age between the groups (BC, 59.6 ± 8.7 yrs; EC, 60.3 ± 7.7 yrs). However, endometrial cancer patients were more likely to be obese (BMI: BC: 29.7 ± 7.9 kg/m^2^; EC: 34.2 ± 8.0 kg/m^2^; p<0.05). On average, neither group of women had subjective sleepiness, with an average ESS less than 10 [12], and there was no significant difference between the groups (p = 0.67). STOP-BANG scores were significantly higher in women with endometrial cancer (p<0.04), while 46% of patients with breast cancer and 57% of patients with endometrial cancer had a STOP-BANG score greater than or equal to 3 (Table 1).

**Table 1:** Anthropometric data, ESS and STOP-BANG questionnaire scores for breast and endometrial cancer patients.

	Breast Cancer (n = 50)	Endometrial Cancer (n = 37)	P value
Age (yrs)	59.6±8.7	60.3±7.7	0.68
Height (cm)	159.7±6.6	158.3±8.0	0.38
Weight (kg)	75.5±19.2	86.4±23.7*	0.02
BMI (kg/m^2^)	29.7±7.9	34.2±8.0*	0.01
Neck circumference (cm)	35.4±3.6	37.1±4.1	0.052
Epworth Sleepiness Scale (au)	6.4±4.4	6.8±4.7	0.67
STOP BANG Score (au)	2.6±1.5	3.4±1.7	0.04
STOP BANG≥3 (n)	23 (46%)	21 (57%)	0.27

Data are mean±standard deviation.

* = p<0.05 for independent samples t-test or Fishers exact test.

### Cancer histopathology

#### Breast cancer

The majority of patients had a history of a ductal malignancy (42/50, 84%), and most cases for which data were available were positive for progesterone (40/46, 87%) and/or estrogen receptors (42/45, 93%). Only two women had triple negative breast cancer (ie were negative for HER2, estrogen receptor or progesterone receptor) (Table 2a).

**Table 2:** (a) Histopathological data for breast cancer patients. (b) Histopathological data for endometrial cancer patients.

**(a)**	
**Histopathological type**	Number (%)
	n = 50^a^
Ductal	42 (84%)
Lobular	7 (14%)
Other	1 (2%)
**Immunohistochemistry**	
Estrogen receptor (n = 45)	
ER-positive	42 (93%)
ER-negative	3 (7%)
Not known (n = 5)^b^	
Progesterone receptor (n = 46)	
PR-positive	40 (87%)
PR-negative	6 (13%)
Not known (n = 4)^b^	
HER2 receptor (n = 42)	
HER2-positive	4 (19%)
HER2 negative	38 (90%)
Not known (n = 8)^b^	
Triple negative	2 (5%)
Ki67 (n = 43)	
Ki67≤15%	18 (42%)
Ki67>15%	25 (50%)
Not known (n = 7)^b^	
Mitotic Index (n = 41)	
<6%	21 (51%)
6–10%	4 (10%)
>10%	16 (39%)
Not known (n = 9)^b^	
**Nottingham histological grade (n = 48)**	
Grade 1	13 (27%)
Grade 2	17 (35%)
Grade 3	18 (38%)
Not known (n = 2)^b^	
**(b)**	
**Histopathological Subtype**	Number (%)
	n = 37^c^
Endometrioid	32 (86.5%)
Serous	0
Clear Cell	1 (2.7%)
Mixed	2 (5.4%)
Other	2 (5.4%)
**Grade (n = 36)**	
Grade 1	23(64%)
Grade 2	9 (25%)
Grade 3	4 (11%)
Not known (n = 1)^d^	
**Immunohistochemistry**	
Estrogen Receptor (n = 23)	
ER-positive	23 (100%)
ER-negative	
Unknown (n = 14)^d^	
Progesterone Receptor (n = 24)	
PR-positive	23 (96%)
PR-negative	1 (4%)
Unknown (n = 13)^d^	
TP53 Staining (n = 34)	
TP53	
wild type	6
abnormal	1
no staining	27
Unknown (n = 3)^d^	
**FIGO Stage (n = 35)**	
Ia	22 (63%)
Ib	4 (11%)
II	4 (11%)
IIIa	1 (3%)
IIIb	0 (0%)
IIIc	3 (9%)
IV	1 (3%)
Unknown (n = 2)^d^	

HER2: (Human Epidermal growth factor 2), Ki67: a cell proliferation marker, Triple negative: (negative for oestrogen, progesterone or HER2).

^a^ Total, n = 50 unless indictated.

^b^ cases with missing data not included in analysis.

^c^ Total, n = 37 unless indictated.

^d^ cases with missing data not included in analysis.

#### Endometrial cancer

The majority of women in this cancer group had a histopathological diagnosis of Grade 1 (FIGO Stage 1 or II) endometrioid cancer. The majority of tumours were positive for progesterone and/or estrogen receptors, and seven of the tumours stained for TP53 (Table 2b).

#### Cancer treatment

*Treatment for BC*. 1) 13 (26%) of patients had a mastectomy, the remainder had a lumpectomy; 2) 39 (78%) of patients had adjuvant radiotherapy, 3) 27 (54%) had chemotherapy, and 4) 34 (68%) of patients were on a current hormonal treatment (either tamoxifen or an aromatase inhibitor).

*Treatment for EC*. 1) all patients underwent a hysterectomy, 2) 15 (40.5%) of patients received adjuvant radiotherapy, and 3) 5 (13.5%) patients received adjuvant chemotherapy.

#### Sleep architecture

Total sleep time and sleep efficiency were not significantly different between the two groups. However, women with breast cancer had a longer sleep latency duration, less slow wave sleep and a greater proportion of rapid eye movement sleep than the endometrial cancer group (Table 3).

**Table 3:** Group average data for sleep architecture measures, hypoxia metrics and sleep disordered breathing (SDB) severity metrics for the two cancer cohorts.

	Breast Cancer (n = 50)	Endometrial Cancer (n = 37)	P value
**Sleep Architecture**			
Total Sleep Time (mins)	359.8±67.6	366.3±67.3	0.67
Sleep Latency (mins)	31.8±32	19.3±17.9*	0.03
Stage 1 (%)	7.8± 6.3	10.1 ±9.7	0.18
Stage 2 (%)	49.9 ± 9.7	48.5± 11.6	0.55
Stage 3 (%)	20.0 ± 5.2	23.6 ±8.2*	0.02
REM (%)	21.1±6.9	16.6±5.7*	0.02
WASO (minutes)	83.6 ±58.8	95.5 ±51.4	0.33
Arousal index (events/ hr)	26.8±14.9	28.3 ±17.5	0.66
Sleep Efficiency (%)	76.0±13.4	76.6±12.1	0.96
**SDB and Hypoxia Metrics**			
Average SpO2 awake	95.6±1.3	94.6±1.9*	0.003
Average SpO2 during sleep	94.8±2.1	93.3±2.5*	0.006
AHI (events/hr)	21.2 (7.3–36.9)	15.7 (10–33.5)	0.733
AHI Categories n (%)			0.00
AHI <5 events/hr	6 (12)	6 (16)	
5 to <15 events/hr	15 (30)	10 (27)	
15 to <30 events/hr	13 (26)	9 (24)	
>30 events/hr	16 (32)	12 (32)	
RDI (events/ hr)	23.8 (7.9–38.9)	18.9 (10–34)	0.44
Central apnea index(events/hr)	0.0 (0.0–0.2)	0.2 (0.0–0.5)	0.03
ODI (3%)	7.3 (1.9–18)	10.0 (2.4–20.9)	0.6
TST <90%	0.45 (0–3.8)	2.4 (0.15–9.7)	0.25
Nadir SpO2(%)	86 (78–91)	84 (75.5–89)	0.4

Data are mean±standard deviation, or median, interquartile ranges (AHI, RDI, ODI, TST<90%, Nadir SpO2(%)) or n (%) for AHI categories,

* = p<0.05.

### Hypoxia metrics and sleep disordered breathing

**Fig 1:**
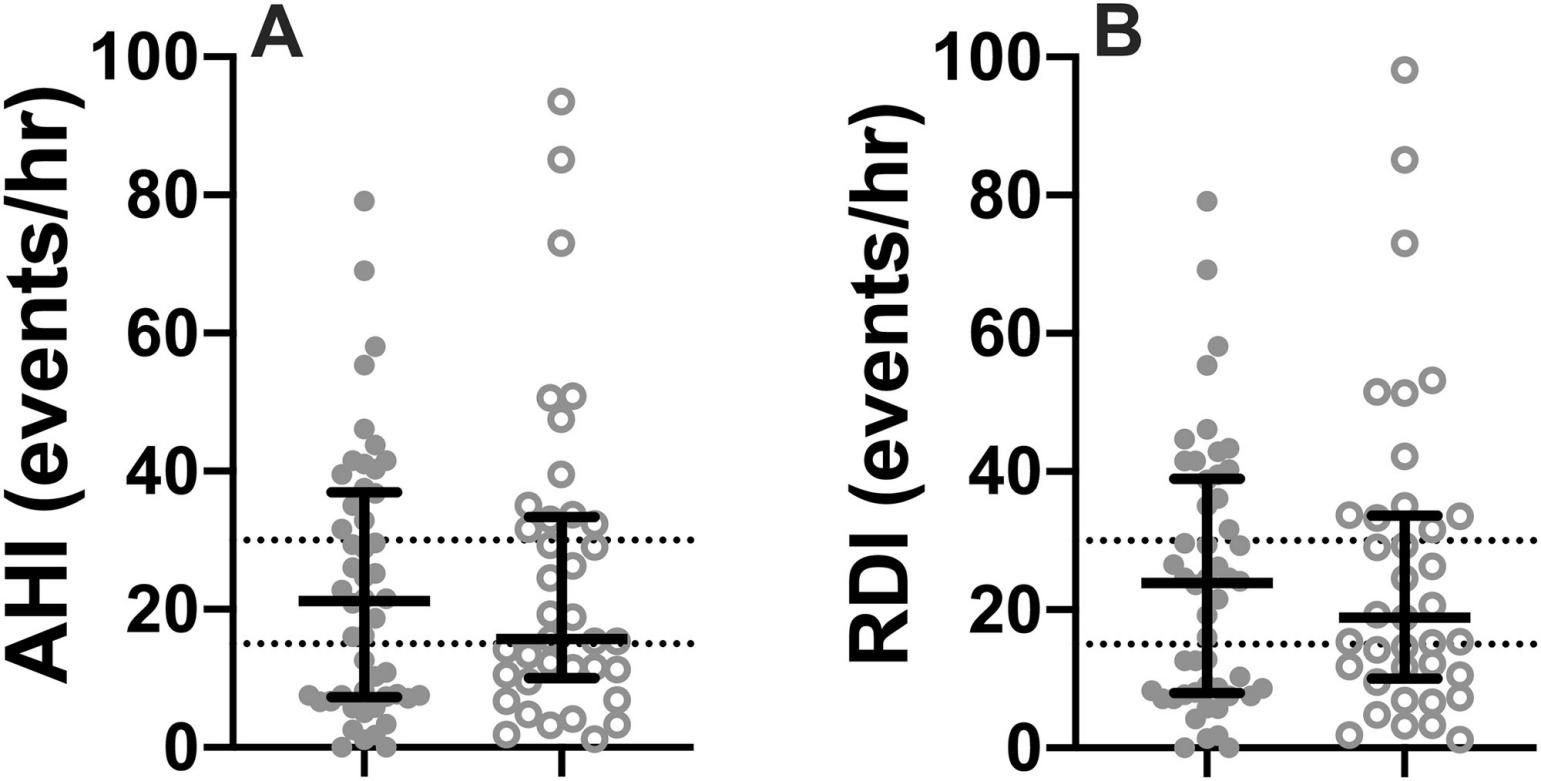
Individual data for breast cancer and endometrial cancer patients for: (A) apnea hypopnea index (AHI) and (B) respiratory disturbance index (RDI). Bars represent median and interquartile range, dotted lines are at 15 events/hour (moderate OSA) and 30 events per hour (severe OSA), closed circles are breast cancer patients, open circles are endometrial cancer patients.

The endometrial cancer group had significantly lower average oxygen saturation levels during wakefulness. There were no significant differences between the two cancer groups for the severity of sleep disordered breathing (as measured by AHI and RDI). Measures of nocturnal oxygen saturation (ODI, TST<90%) also did not differ between the groups. However, 58% of the breast cancer patients, and 57% of endometrial cancer patients had an AHI >15 events per hour, while 32% of the breast cancer cohort and 32.4% of the endometrial cancer cohort had an AHI >30 events per hour. Women with endometrial cancer had a higher central apnea index (p<0.03) (see Table 3, Fig 1).

### Relationships with tumour histopathological characteristics

**Fig 2:**
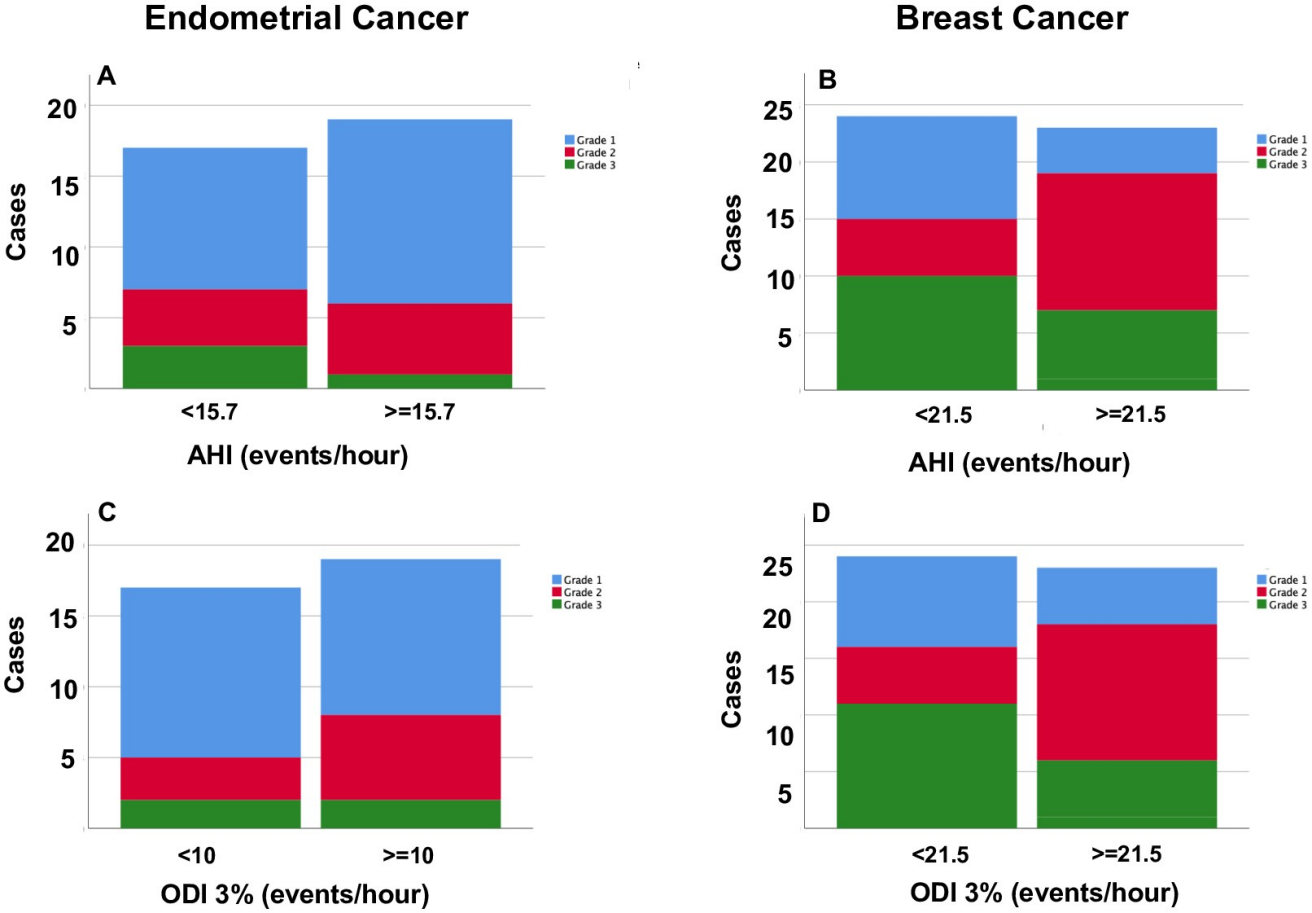
Frequency distributions for histopathological grades for endometrial cancer (Grade 1–3 (11]) (A, C) and breast cancer (Nottingham Grade 1–3) [10] (B, D), plotted against the OSA categorical variables determined from median values for apnea hypopnea index (AHI events/hour) and oxygen desaturation index (ODI 3% events/hour) for each of the cancer patients. There were no significant differences in the distribution of histological grades (all p>0.1, Fisher’s exact test).

For both breast and endometrial cancer patients, there were no significant differences in the prevalence of any histopathological marker between the two sleep disordered breathing severity sub-groups (based on any polysomnography metric) (all p>0.09, Fisher’s Exact test, see Table 4, Fig 2).

**Table 4:** Distribution of histopathological characteristics across the categorical variables for sleep disordered breathing for (a) breast cancer and (b) endometrial cancer.

**(a)**								
	AHI (events/hour)		ODI (events/hour)		TST<90 (%)		RDI (events/hour)	
	<21.5	≥21.5	<7.3	≥7.3	<0.5	≥0.5	<23.8	≥23.8
Estrogen receptor								
(n = 45)								
ER-positive (n)	21	21	21	21	22	20	21	21
ER-negative (n)	2	1	2	1	2	1	2	1
p	0.7		0.7		0.6		0.7	
Progesterone receptor								
(n = 46)								
PR-positive (n)	22	18	23	17	23	17	22	18
PR-negative (n)	2	4	1	5	1	5	2	4
p	0.5		0.1		0.1		0.3	
HER2 receptor (n = 42)								
HER2 positive (n)	3	1	3	1	3	1	3	1
HER2 negative (n)	20	18	20	18	20	18	20	18
p	0.2		0.2		0.2		0.2	
Ki67 staining (n = 43)								
Ki67 ≤15%	9	9	8	10	9	9	8	10
Ki67 >15%	14	11	15	10	14	11	15	10
p	0.4		0.2		0.4		0.2	
Mitotic Index								
<6%	11	10	10	11	11	10	11	10
6–10%	2	2	2	2	2	2	2	2
>10%	9	7	10	6	8	8	9	7
P	0.8		0.5		1.0		0.8	
Nottingham Grade								
Grade 1	9	4	8	5	9	4	9	4
Grade 2	5	12	5	12	6	11	5	12
Grade 3	10	8	11	7	9	9	10	8
p	0.2		0.1		0.3		0.2	
**(b)**								
	AHI (events/hour)		ODI (events/hour)		TST90 (%)		RDI (events/hour)	
	<15.7	≥15.7	<10	≥10	<2.4	≥2.4	<8.9	≥8.9
Grade (n = 36)								
Grade 1 (n)	10	13	12	11	11	12	5	18
Grade 2 (n)	4	5	3	6	5	4	1	8
Grade 3 (n)	3	1	2	2	2	4	1	3
p	0.6		0.8		1.0		0.3	
FIGO Stage (n = 35)								
I	12	14	14	12	14	12	6	20
II-IV	5	4	3	6	4	5	1	8
p	0.8		0.7		0.8		0.3	

P values are results of Fisher’s exact test (2-sided significance).

## Discussion

This is the first study to compare polysomnography findings for women across two specific post-treatment cancer patient groups, breast and endometrial cancer. In doing so, we also report the first study to examine the prevalence of OSA in women diagnosed with endometrial cancer; a female cancer cohort with the OSA-shared risk factor of obesity.

The major finding of this study is that more than half of the women in each of these cancer patient groups were found to have at least moderate OSA (AHI>15 events/hr), and nearly one third had severe OSA (AHI>30 events/hr). There were no differences between the two cancer patient groups in terms of the overall severity of OSA, although women with endometrial cancer had slightly lower average oxygen saturation levels, both awake and asleep. Excessive sleepiness was not a feature of either of these cohorts. There were no associations between measurements of OSA severity and any tumour histopathological feature or malignancy grade within either cohort.

### Prevalence of sleep disordered breathing in women with a history of cancer

In this study of female cancer, OSA prevalence was greater than that reported for community-based population studies. Based on previous data [16], and adjusted for increasing obesity prevalence [17] it has been estimated that 9% (confidence interval 7–11%) of women between 50–70 years have moderate- severe OSA (AHI>15 events per hour). The more recent HypnoLaus study suggests a population prevalence of 23.4% for moderate-severe OSA (AHI>15 event/hr) in women with an average age of 58 years [18]. The prevalence of OSA in both female cancer cohorts is higher than population prevalence; however, similar to that reported in obese postmenopausal women of 67% with an RDI greater than 10 events per hour [19]. Based on the STOP-BANG score, at least half of the patients were at risk for OSA, however, age and obesity would have resulted in the majority of patients scoring at least 1 on this questionnaire. This increased prevalence compared to the population is most likely a consequence of shared risk factors for breast cancer, endometrial cancer and OSA of obesity and postmenopausal status for both groups. Endometrial cancer is one of the cancers most strongly associated with obesity, however obesity is also a risk factor for post menopausal breast cancer [7].

The prevalence of OSA in breast cancer patients reported in the present study is higher than previously reported. A study of 83 breast cancer patients in Spain, with an average age of 48.8 years, estimated moderate-severe OSA prevalence (AHI>15 events/hr) at only 13.3% [6]. That study used limited channel home sleep studies and nearly half of the women were pre-menopausal, both of which may have reduced the measured prevalence. In another study of breast cancer patients after treatment, which specifically excluded women with a history of snoring but used polysomnography, there were no women with moderate to severe OSA [20]. We could find no previous polysomnography study reports in women with endometrial cancer.

The OSA prevalence in the present study was such that nearly one third of both cancer cohorts reached a severity level for sleep disordered breathing that warranted treatment, and based on STOP-BANG screening, almost half the breast cancer patients and two thirds of the endometrial cancer patients were at high risk of having OSA. A lack of recognition and diagnosis of sleep disordered breathing in women is an issue that has been identified by others [21]. The reasons for this underdiagnosis may be a consequence of an absence of either subjective or objective sleepiness, as has been reported by others [22, 23], and which was also present in both our female cancer patient groups. The reasons for this high prevalence in these female cancer patients are not clear. It is most likely related to the presence of shared risk factors (obesity, age, postmenopausal status) for OSA and both endometrial and breast cancer in these cohorts [7, 24–26]. However we are cognisant that there is potential for patient self-selection bias in a volunteer cohort sleep study, and not all patients who met criteria for a sleep study in this study volunteered to have one, likely further enriching the sample for women with OSA. A high OSA prevalence may also be a reflection of an underlying association between OSA and cancer incidence [1, 2, 27] which has been reported to be particularly high in women [28]. Some epidemiology studies have also reported that OSA is a specific risk factor for breast cancer [29, 30], although other studies have failed to demonstrate this relationship [27].

#### Generalisability and clinical relevance

All women in this study were well known to the health system and were receiving specialised care for their cancer, despite this, their OSA status remained undiagnosed. Irrespective of the potential interaction between OSA and cancer incidence and mortality, there are important health, quality of life and clinical management issues associated with undiagnosed OSA. Epidemiological studies suggest that in women associations between OSA and hypertension [31], and cardiovascular disease [32, 33] are stronger than in men. Similarly OSA in women is associated with systemic inflammation at lower levels of sleep disordered breathing than men [34]. Finally, recent data demonstrates that unrecognised severe OSA (AHI>30 events/hr) increases the risk of perioperative cardiovascular events [35]. We did not collect data on cardiovascular risk in this study, however all of the women in this study had undergone surgical procedures as part of their cancer treatment. Identification of OSA in this group of women with cancer is important for both short term management of peri-operative risk, and longer term management of inflammation and cardiovascular disease.

### Relationships between metrics of sleep disordered breathing and cancer aggressiveness

We were unable to demonstrate any relationships between histopathological features of cancer aggressiveness and any metric of sleep disordered breathing in either group, although we recognise that the cohorts are relatively small for analysis of multiple clinical variables. In particular, within the breast cancer cases, few cases were negative for estrogen, progesterone and HER-2 receptors. Similarly, small numbers of endometrial cancer cases stained for TP53, or were negative for reproductive hormones.

Risk factors for specific cancers vary, and existing evidence suggests OSA pathophysiology may not interact with all cancers. Our findings are similar to recent findings in 83 women with breast cancer [6]. These women were younger than our cohort, with a lower prevalence of moderate-severe sleep disordered breathing, although grade and histopathological subtype of the cancers present were similar [6]. The limitations on the present study for finding an interaction include that participant numbers, particularly for endometrial cancer patients, were not powered to investigate interactions between OSA and cancer aggressiveness, nor was there sufficient hetereogeneity in the cancer histopathology to demonstrate a signal. Within the endometrial cancer patients, the majority of the subject group were low grade endometrioid tumours. However, our findings in the breast cancer patients are in agreement with previous reports [6]. There have been no previous investigations of relationships between endometrial markers of cancer aggressiveness and sleep disordered breathing, and our negative findings are the first to be reported.

A lack of molecular subtyping of tumours may also have limited our ability to demonstrate significant interactions between tumours and sleep disordered breathing. Endometrial cancer can be divided into four molecular subtypes [36], which differ despite similar histopathological appearances. Similarly, there is a great deal of heterogeneity in breast cancer molecular subtypes [37]. Systemic fluctuating oxygen levels, or intermittent hypoxia, has been demonstrated to alter genotypes and phenotypes of epithelial cell lines, with reduction in TP53 expression (a tumour suppressor gene) and increasing the expression of hypoxic inducible factor alpha (HIF1a) [38]. Intermittent hypoxia is a feature of both groups of cancer patients in this study, and molecular subtyping of the tumours may provide more insight into interactions with OSA.

Finally, this was a study of cancer patients after treatment, and sleep studies were performed 12 months (breast cancer) and 3 months (endometrial cancer) after initial diagnosis. Severity of OSA measured at this time point may not be reflective of OSA severity prior to the cancer diagnosis.

### Sleep architecture and OSA differences between women with cancer

There were differences between the two groups in terms of sleep architecture, with women with endometrial cancer having less REM sleep, more slow wave sleep and a shorter sleep latency. Apart from obesity, there were no significant demographic differences between the two groups. Obesity is reported to reduce slow wave sleep with no effect on REM sleep in men [39], although others have reported no relationship between BMI and SWS and REM in larger population samples [40]. Thus, reasons for differences in sleep architecture are not clear. Women with breast cancer complain of poor sleep, and up to 60% of women highlight this as an issue after breast cancer treatment [41]. There are few studies in women with endometrial cancer, however, one study found that 61% of women with endometrial cancer had subjective poor sleep quality [42]. Postmenopausal status may have influenced sleep architecture. A recent extensive review of polysomnographic studies in women during the menopausal transition, concluded there are few consistent effects of menopausal stage on sleep architecture [43]. However, slow-wave sleep is more prevalent in a large population samples of post versus pre-menopausal women and postmenopausal women on hormone replacement with oestrogen and progesterone also have less slow wave sleep [43]. Differences between the two cancer groups in sleep architecture in the present study may be related to differences in reproductive hormone levels.

There were no significant differences between the two groups of female cancer patients in terms of the severity of OSA, although average oxygen saturation awake and asleep was lower in the endometrial cancer patients. This likely reflects the increased obesity in the endometrial cancer patients. Similar prevalences and severity of OSA between breast and endometrial cancer patients are likely reflective of the age and obesity risk factors, for both cancer and OSA, that were present in both groups.

### Strengths and limitations of this study

This is the first prospective study of endometrial cancer patients, and the first prospective study of breast cancer patients to use gold standard laboratory polysomnography to assess OSA severity and examine relationships with cancer histopathology. Although limited channel studies have been shown to result in similar clinical outcomes [44], there are differences in the assessment of OSA severity, which may be important. We have chosen to study two female cancer cohorts, allowing for comparisons between the two groups.

Study limitations include the small sample sizes, which may have resulted in an incorrect estimation of prevalence of sleep disordered breathing in the cancer cohorts. Similarly small sample sizes may have impacted on our ability to find any relationships between cancer characteristics and OSA, as has a lack of molecular subtyping of the tumours. The timing of the sleep studies were different in each group because of the different therapies each cancer group underwent. At 12 months, women with breast cancer have been demonstrated to have returned to baseline in terms of sleep symptoms [45]. There is no data on patients with endometrial cancer, however we chose to study these patients within 3 months of treatment, as the majority had surgery only. For those endometrial cancer patients who had radiotherapy or chemotherapy, there was a minimum of 7 months after treatment. These differences in timing may have impacted OSA prevalence or severity, although we did not find any differences between the groups.

## Conclusion

Women with breast or endometrial cancer have a high prevalence of moderate-severe OSA that may go unrecognised, potentially due to a lack of sleepiness symptoms and an atypical presentation. While we found no relationships between the severity of sleep disordered breathing and histopathological characteristics of cancer in these women, or any differences in OSA severity between the study groups, OSA likely poses an increased anesthetic and surgical risk for these patients during their treatment phase, and should also be considered as a potential contributor to on-going quality of life issues that may be experienced by breast and endometrial cancer patients in the post-treatment phase.

## Supporting information

S1 Appendix (DOCX)
