# Supplementary material for: Increased prevalence of obstructive sleep apnea in women diagnosed with endometrial or breast cancer
Source: PLoS One. 2021 Apr 7;16(4):e0249099. doi: 10.1371/journal.pone.0249099 (PMC8026058; doi:10.1371/journal.pone.0249099)
Supplement: S1 Appendix — (DOCX) [file pone.0249099.s001.docx]

**S1 Appendix.**

Treatment for BC- 13 (26%) of the patients had a mastectomy, the remaining patients had a lumpectomy. 39 (78%) of the patients had adjuvant radiotherapy and 27 (54%) had chemotherapy. 34 of the patients were on a current hormonal treatment (either tamoxifen or an aromatase inhibitor.

Treatment for EC was hysterectomy in all women. 15 (40.5%) of the women had adjuvant radiotherapy, and 5 (13.5%) women had adjuvant chemotherapy.
